# Bone Marrow Immunity and Myelodysplasia

**DOI:** 10.3389/fonc.2016.00172

**Published:** 2016-07-20

**Authors:** Claude Lambert, Yuenv Wu, Carmen Aanei

**Affiliations:** ^1^Immunology Laboratory, Pole de Biologie-Pathologie, University Hospital of St Etienne, St Etienne, France; ^2^Haematology Laboratory, Pole de Biologie-Pathologie, University Hospital of St Etienne, St Etienne, France

**Keywords:** bone marrow, monocytes, macrophage, myelodysplasia, T cell

## Abstract

Myelodysplastic syndrome (MDS) is characterized by an ineffective hematopoiesis with production of aberrant clones and a high cell apoptosis rate in bone marrow (BM). Macrophages are in charge of phagocytosis. Innate Immune cells and specific T cells are in charge of immunosurveillance. Little is known on BM cell recruitment and activity as BM aspirate is frequently contaminated with peripheral blood. But evidences suggest an active role of immune cells in protection against MDS and secondary leukemia. BM CD8^+^ CD28^−^ CD57^+^ T cells are directly cytotoxic and have a distinct cytokine signature in MDS, producing TNF-α, IL-6, CCL3, CCL4, IL-1RA, TNFα, FAS-L, TRAIL, and so on. These tools promote apoptosis of aberrant cells. On the other hand, they also increase MDS-related cytopenia and myelofibrosis together with TGFβ. IL-32 produced by stromal cells amplifies NK cytotoxicity but also the vicious circle of TNFα production. Myeloid-derived suppressing cells (MDSC) are increased in MDS and have ambiguous role in protection/progression of the diseases. CD33 is expressed on hematopoietic stem cells on MDS and might be a potential target for biotherapy. MDS also has impact on immunity and can favor chronic inflammation and emergence of autoimmune disorders. BM is the site of hematopoiesis and thus contains a complex population of cells at different stages of differentiation from stem cells and early engaged precursors up to almost mature cells of each lineage including erythrocytes, megakaryocytes, myelo-monocytic cells (monocyte/macrophage and granulocytes), NK cells, and B cells. Monocytes and B cell finalize their maturation in peripheral tissues or lymph nodes after migration through the blood. On the other hand, T cells develop in thymus and are present in BM only as mature cells, just like other well vascularized tissues. BM precursors have a strong proliferative capacity, which is usually associated with a high risk for genetic errors, cell dysfunction, and consequent cell death. Abnormal cells are prone to destruction through spontaneous apoptosis or because of the immunosurveillance that needs to stay highly vigilant. High rates of proliferation or differentiation failures lead to a high rate of cell death and massive release of debris to be captured and destroyed (1). Numerous macrophages reside in BM in charge of home-keeping. They have a high capacity of phagocytosis required for clearing all these debris.

## Immunity in Bone Marrow

**Figure 1:**
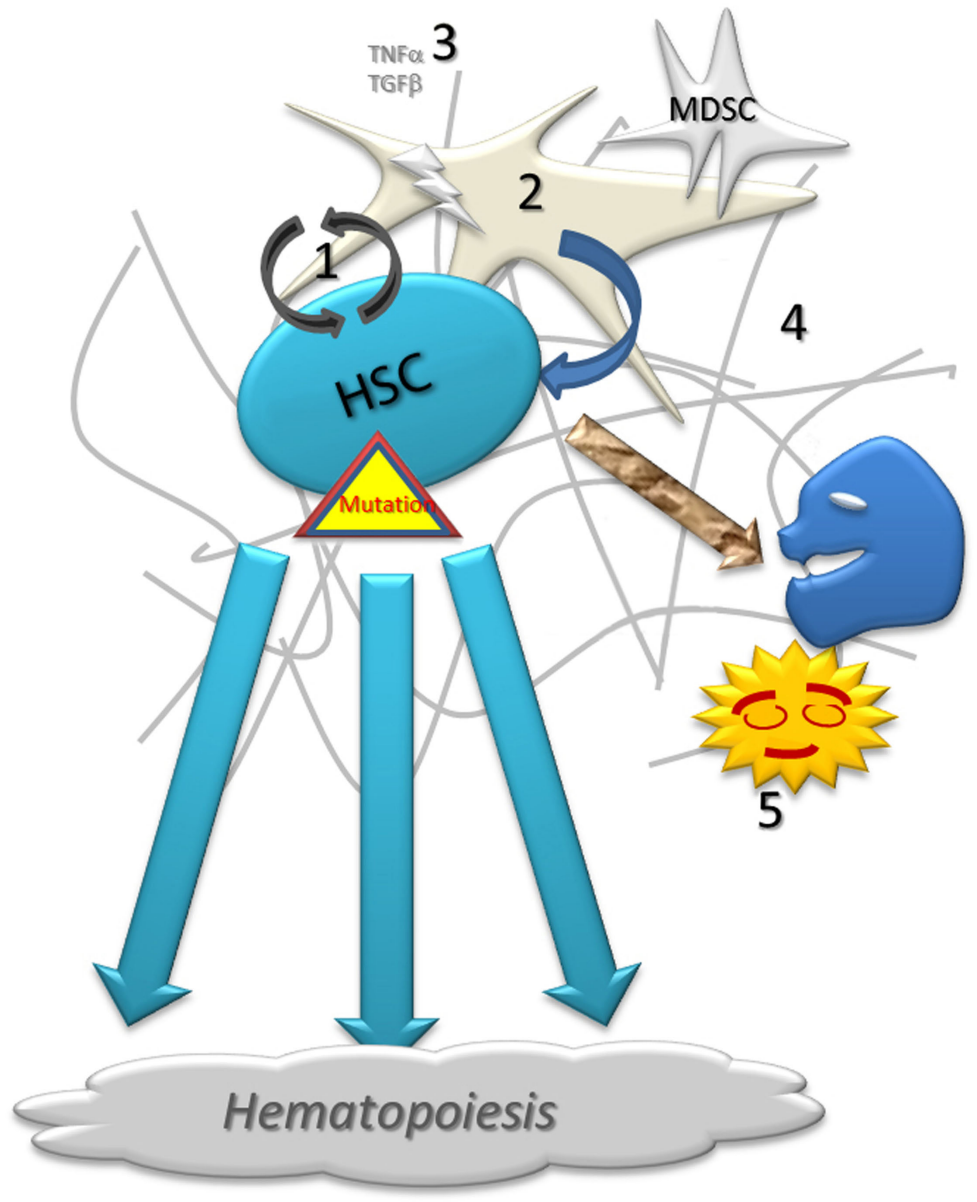
**Schematic representation of normal hematopoiesis**. Normal hematopoietic stem cell (1) self-renew with the close support of stroma (2) and growth factors (3) on the fibrotic matrix (4). A low proportion of cells fails during the proliferation and differentiation processes. Abnormal cells are destroyed by apoptosis or under immune surveillance, being phagocytized by resident macrophages (5).

The immune effectors penetrates into the BM hematopoietic niche with difficulty, and this is probably the reason for which the BM is a predilection site for the metastases from solid tumors where they like to hide and develop (Figure 1) (2). BM bacterial infections are rare. This may be because the BM is deep and bacteria probably have to go through multiple barriers to reach it. But we cannot exclude a role of the immune system in the surveillance and occasional protection against any infection event. In any case, this activity is difficult to evaluate *in situ*. Indeed, BM is easily analyzed, specially with the help of flow cytometry, but BM sampling requires a deep aspiration that is inevitably contaminated by peripheral blood. BM biopsy only gives precious information on anatomical distribution but it is limited in qualitative and quantitative characterization of resident cells.

In general, immunosurveillance plays an important role in maintaining homeostasis and eliminating aberrant clones (3). Deficiencies in immunosurveillance raise the risk of developing tumors. In the same line, some therapeutic strategies have been designed to boost the immunosurveillance for preventing relapse and eventually cure cancer (4). T cells and type I Innate Lymphoid cells (ILC) are the major effectors of specific or innate cytotoxicity. ICL type I includes NK cells. Macrophages closely cooperate with lymphocytes through the production of cytokines and chemokines. Effector lymphocytes are mobile in the blood stream or reside quietly in the lymphoid tissues, far from BM. Lymphocytes are only recruited in the tissues if needed, driven by the gradient of chemokines. For that purpose, they express chemokine receptors and adhesion molecules. A few chemokines receptors or adhesion molecules have some tissue specificity such as CCR7, alpha4-beta7, or CD103 and CLA for Lymph node, mucosae or skin, respectively, while CXCR3 guide cells to inflamed tissues (5). Analyzing the expression of chemokine receptors is a good tool to identify cells specifically recruited in the BM (6) but, unfortunately, no homing receptor specific for BM is identified yet.

Under activation, lymphocytes produce cytokines and build up complex crosstalks between all effectors. Cytokines such as IL-2, IFN-γ, IL-17, TNFα promote inflammation and cytotoxicity while IL-10 or TGFβ downregulated the immune response and promote tissue healing. These mediators are required to set up a good immunosurveillance but are poorly discriminant and can incidentally help pathological cell growth and/or downregulate immune cells. For example, TGFβ promotes healing but is a strong inducer of Regulatory T cells (Treg). Similarly, Myeloid-derived suppressing cells (MDSC) support progenitor proliferation, partly through CD33 receptor (7) but can also support tumor growth by producing growth factors or angiogenic factors (8). MDSCs express CD45, CD33, and CD11b^+^ and derive from monocytes (CD14^+^ CD15^−^) or granulocytes (CD14^−^ CD15^+^) (9). As a matter of fact, granulocyte-derived MDSC was reported to be increased in BM and PB of multiple myeloma (9, 10) and can promote the tumor growth (11) raising interesting therapeutic potentials (12).

## Feeding Activity of the Stroma in Normal Bone Marrow

The process of hematopoiesis is closely related to its micro-environment that includes extracellular matrix, mesenchymal cells, macrophages, etc. The stromal cells are supporting the survival and proliferation of progenitor cells (13, 14). Macrophages are also contributing a lot in this support through direct contact with progenitors or by producing growth and angiogenic factors. Notably, TNFα can promote proliferation of hematopoietic progenitors (15). Again, this support is not discriminant and helps healthy and pathological clones as well, but pathological clones frequently acquire proliferative advantages over normal cells.

Myelodysplastic syndrome (MDS) is a heterogeneous group of acquired clonal disorders of stem cell, altering hematopoiesis efficiency. Stem cell renewal is increased raising the risks for mutations and cell aberrancies, and MSD is associated with a higher risk for the development of Acute Myeloblastic Leukemia (AML). The efficiency of cell differentiation is reduced leading to cytopenia that concerns variably one or several lineages. The microenvironment is progressively damaged and fails in stromal support but induces progressive myelofibrosis (2, 16). This proliferation and maturation disorders are associated with an increased rate of cell death by apoptosis or pyroptosis. The cause(s) of MDS remains unknown. Few genetic or environmental conditions promote MDS (17).

## Possible Role of Microenvironment in MDS

The MDS primary disorder is generally believed to come from the stem cell. However, stem cell behavior is closely related to the stroma, and stromal disorders have also been shown in MDS, even when stromal cells are cultivated in absence of pathological hematopoietic cells. This suggests that the primary disorder could also start with stromal cells (18). However, the micro-environment is certainly disturbed by the pathological clone (19) and particular mesenchymal cells could also be actively recruited by aberrant cells through the production of CCL25 as it has been described in myeloma (20). At the end, experts conclude that the cooperation effect is most probably “mutual and reciprocal” (21). Still remains the possibility that pathological clones and its microenvironment are both victims of a same extrinsic cause that could be infection or toxic agent not yet identified.

## Immune Disorders Associated with Myelodysplastic Bone Marrow

**Figure 2:**
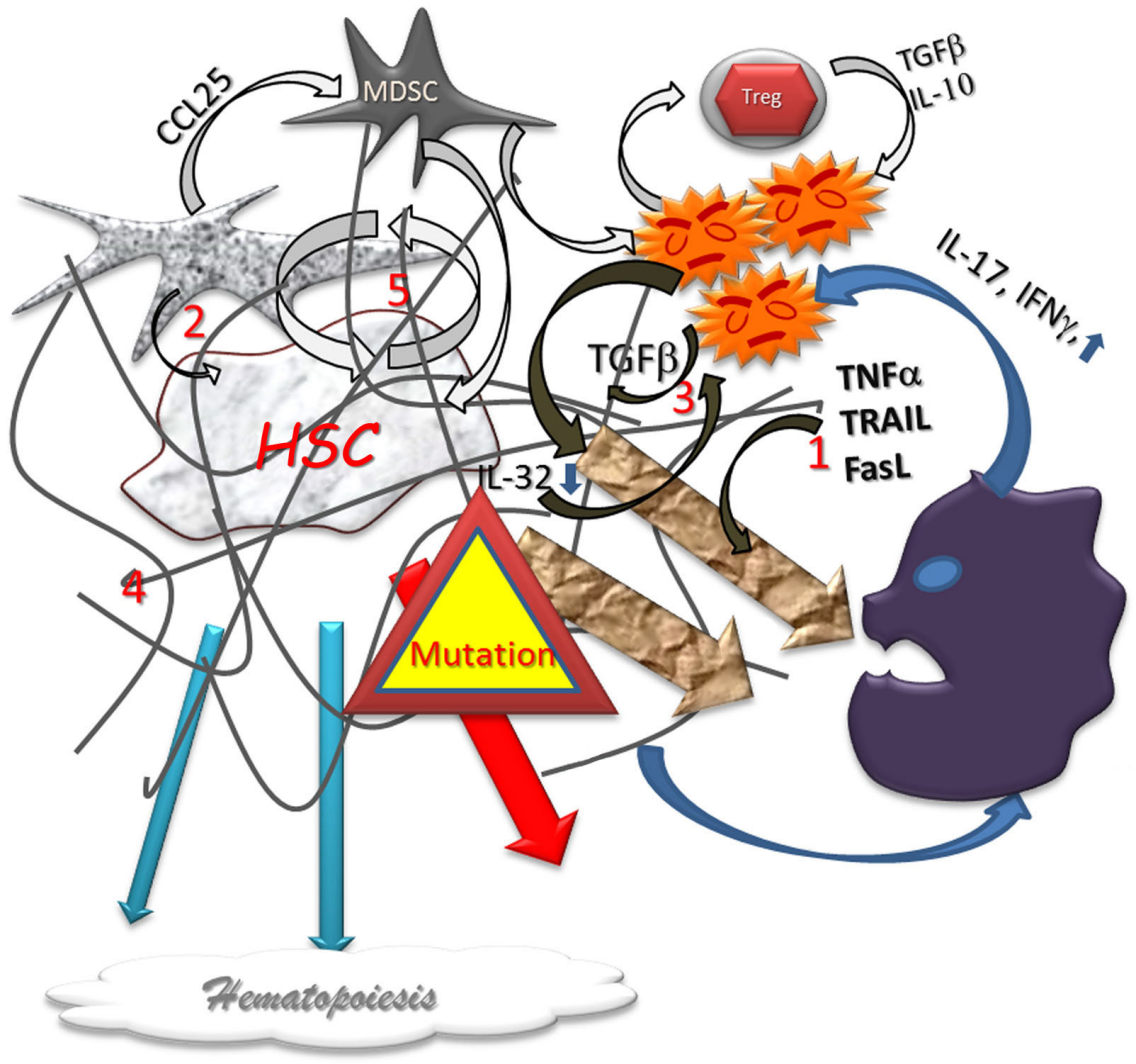
**Dysplastic hematopoiesis**. In myelodysplastic settings, the stem cell function is disturbed by several abnormal conditions such as local inflammation (1) defect in stromal support (2), inappropriate growth factor production (3), myelofibrosis (4), that are conducive to an increased cell renewal (5), and, subsequently, to higher risk of failure and mutations. The production of Alarmins induce a pro-inflammatory vicious circle loop, a cytotoxic immune reaction, and impair the immunomodulatory process. Differentiation in lineages is reduced and eventually aberrant. In addition, the higher level of mutations is responsible for higher home keeping activity with exhaustion.

Despite no extrinsic agent has been implicate, there is evidence of an inflammation process in MDS BM (Figure 2). Numerous studies report there are high productions of TNFα, IFNγ, IL-1α; IL-6, IL-17, TGFβ, using different techniques such as immuno-histology, BM dosages, functional analyses, and even in peripheral blood dosages (22–32). These cytokines can be produced not only by resident macrophages and attracted lymphocytes but also by stromal cells and stem cells. Macrophages are highly engaged in clearing all debris from aborted differentiation of hematopoietic cells (33, 34). Cell damages produce Damage-Associated Molecular Patterns (DAMPs) that are, together with pathogen-associated molecular patterns (PAMPs), strong ligands for Pattern-Recognition Receptors (PRRs). Among the PRRs, the Toll Like Receptor 4 (TLR4) is overexpressed not only by MDS macrophages but also by stem cells and stromal cells (35–37). Among DAMPS, alarmins such as the S100A9 are produced in MDS, and S100A9 was shown to promote pyroptosis and apoptosis (7, 38). A polyclonal T cell infiltrate has also been demonstrated in MDS BM (39), specially CD4^+^ T cells producing IFNγ (40). T cell turnover (41) and apoptosis are elevated and correlated to the cytokine production (42). Effector, cytotoxic (CD8^+^ CD28^−^ CD57^+^) T cells are increased in peripheral blood of MDS patients (43).

## Immune Response Acts Against MDS Progression

The pro-apoptotic cytokines can participate in the elimination of aberrant clones, and progenitors have been shown to express TNF family receptors. NK cells are also directly active in destroying aberrant cells (44). Finally, aberrant phenotypes can induce specific immunity, and T cell infiltrate has been observed, specially CD8^+^ T cells with restricted T cell receptor Vbeta diversity. These cells frequently bear an activated profile (CD25, CD58) and have a high content of cytotoxic proteins (granzyme) as compared to peripheral blood (45). A CD8^+^ T cell proliferation specific for trisomy 8 related aneuploid hematopoietic progenitor cells has been reported (46). The higher CD8^+^ T cell count is associated with a lower count of regulatory T cells (Treg) in BM (47). BM T cells have a distinct functional signature producing high levels of TNF-α, IL-6, CCL3, CCL4, and IL-1RA (48). Note that CCL3 is preferentially produced, and its production is associated with a poor prognostic as reported for CCL5 in AML (49). These evidences strongly suggest that there is an active antigen specific T cell response in BM especially in MDS. It is not clear yet if the T cell specificity recognize an auto-antigen or a neo-antigen related to MDS aberrancies.

## Defect in Immuno Surveillance Favors MDS

In MDS, NK cell number is usually not reduced as compared to healthy donors (50, 51) although it can be low in as much as 20% of patients, independently of T lymphopenia. NK deficiency is directly related to a MDS with poor prognosis such as refractory anemia with excess blasts (52). However, a NK functional deficiency has been reported with a decrease in their content of perforin and granzyme and a defect in production of IFNγ (50,  53, 54). The NK functional deficiency is also associated with a high risk of MDS (55). IL-32 is produced by several cells under stimulation by TNFα or IL-1. NK cells capacity for production of IL-32 is reduced in MDS (56) and as well as the expression of the NK activating receptor (NKG2D). This reduced capacity was associated with the NK functional deficiency (50, 57). The MDS patients are frequently old. The NK activity is also declining with aging, making the NK deficiency due to the diseases even worse (55). Therapeutic strategies forcing NK cells to recognize of CD33^+^ MDS progenitors using bispecific antibodies have been proposed (58). On the other hand, IL-2 or IL-12 therapeutic trials have not shown any significant clinical benefit in MDS (53). Another ILC, the invariable NKT cells, have also been reported to be reduced in MDS (59).

Peripheral Treg are increased in MDS with more than 5% of blasts in accordance with the disease progression (27, 45, 60). This is even more clear if we consider the effector memory Treg, which have higher regulatory activity (61).

Accordingly, some immunodeficiencies favor MDS. The “Monomac syndrome” is a sporadic primary immunodeficiency due to a GATA2 mutation. It associates severe monocytopenia (mean value 10 cells/μL) and possibly B and/or NK and/or dendritic cell deficiencies. Monomac syndrome is frequently revealed by severe infections with atypical agents like non-tuberculous mycobacteria, papillomavirus, or fungi (62). Almost half of these patients develop a MDS or an AML. But it is not clear whether the evolution to MDS is related to the progenitor abnormalities or to the defect of immunodeficiency. In any case, Monomac-related MDS is not really typical as it shows poorer cellularity and prominent reticulin myelofibrosis (63). Few more immunodeficiencies are associated with MDS (64) in which it is difficult to say whether the primary genetic mutations or immunodeficiency is responsible for MDS emergence.

## Immune Disorders Participating to MDS Physiopathology

Few cytokines produced in MDS BM inflammation are strong inducer of apoptosis and could aggravate the MDS-related cytopenia (Figure 2). TNFα (65) and other related cytokines such as TRAIL or FAS-ligand (31, 66) are overproduced in MDS. The dosage of TNFα is correlated with the severity of cytopenia (19). These pro-apoptotic cytokines can also induce damages to the normal progenitors (67, 68) and differentiated cells. Indeed, the overall, neutralization of these TNF-related cytokines was shown to improve hematopoiesis (42, 66), and MDS has been reported in TNFα overproducing transgenic mice (69). Pro-cytotoxic CD4^+^ T cell also has collateral cytotoxic effects participating into the cytopenia. These effects could be partially reduced with treatment (Cyclosporin A) that reduces Th1 activity (40) and cell turnover (41). The TGFβ over production has been related to myelofibrosis in MDS and is associated with poor prognosis (7, 70, 71).

In MDS, MDSCs was reported to be increased too and to produce pro-cytotoxic cytokines (72). MDSC could also have direct suppressive action on stem cell throughout the CD33 binding (72).

## MDS Can Impair the Immune Response

Myelodysplastic syndrome induces immunological disorders especially on the monocytic and myelocytic lineages partly due to an inadequate production of cytokines. Maturation disorder and high apoptosis rate are responsible for phagocytosis exhaustion (73). Peripheral cytopenia is frequent except for NK cells (74). The numbers of B cells or their precursors are strongly reduced but B cell dysplasia and defect in immunoglobulin production is not considered as a major issue in MDS (75). Peripheral T cells are disturbed too with a skewing of its Vbeta repertoire (45). T cells have preferentially the pro-inflammatory profiles Th22 and Th17, at least at an advanced stage of the disease (27).

Myelodysplastic syndrome can be associated with an increased risk for auto-immunity (76) which can, in return, participate in the cytopenia. Autoimmune antibodies against erythrocytes have been shown in aplastic anemia (77) beside auto-antibodies against MDS neo-antigens due to genetic mutations (78). T cell auto-reactivity can also play a role in cytopenia and could be partly responsible for the reduction of Vβ repertoire (45). A few cases of Lymphoproliferative syndrome associated with MDS has also been described (79).

**Figure 3:**
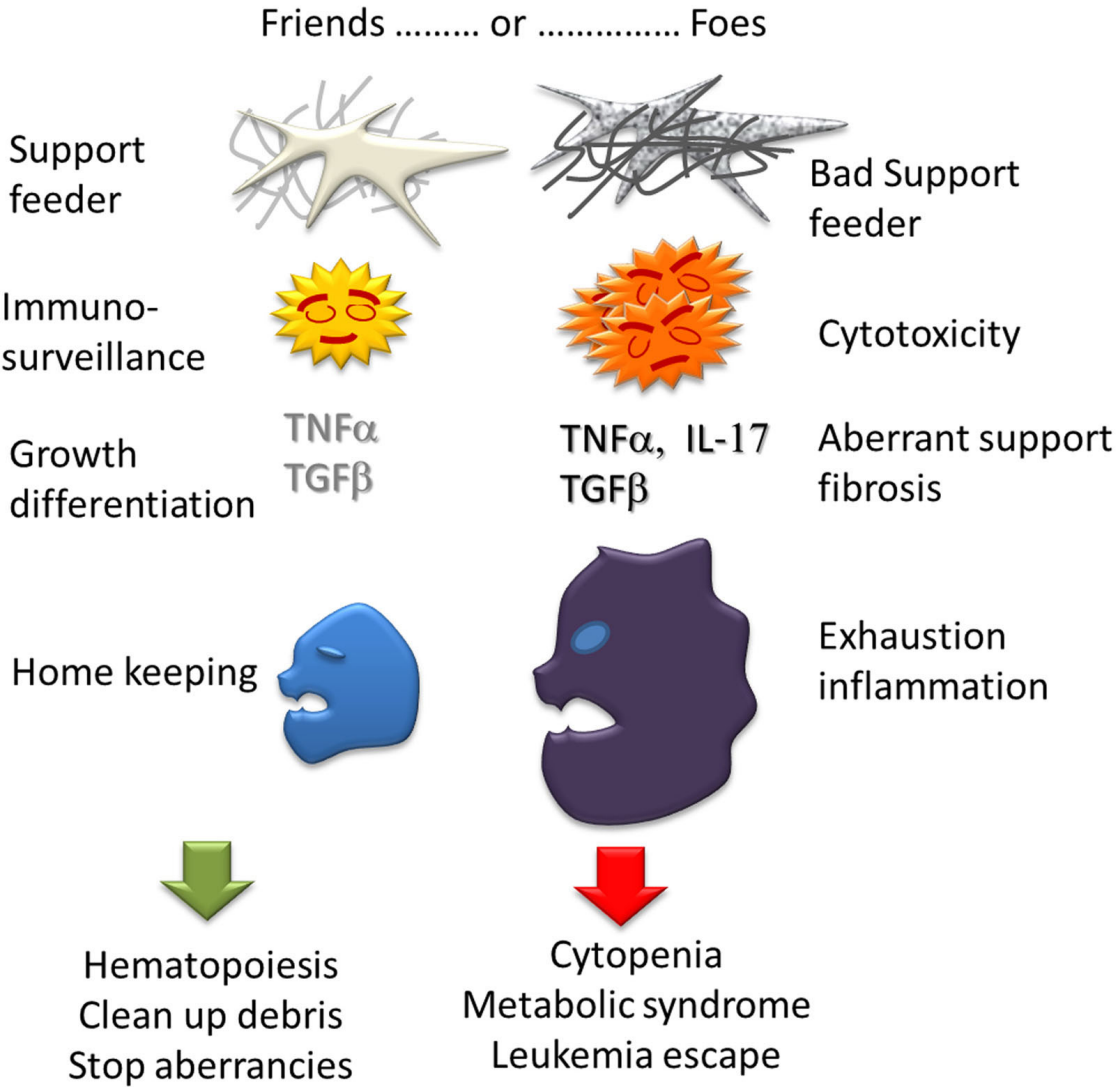
**Ambiguous role of Immunity in MDS**. Each bone marrow component can have actions either good, supporting hematopoiesis and homekeeping in homeostasia, or bad: cytopenia, myelofibrosis, and inflammation in dysplasia.

In conclusion (Figure 3), there are many evidences that immunity plays a role in MDS. However this role is complex and ambiguous. An immunity is expected to prevent or delay the emergence of pathological clones through innate antitumor activity or specific recognition of neo-antigens but it is most probable the MDS clones must find a way to escape the immunosurveillance as solid tumors do. An inflammatory status in BM can be a cause of consequence of the disease. Chronic inflammation could support immunity but also has systemic effect worsening a metabolic syndrome. Exhaustion and immunodeficiencies can favor MDS and AML. On the other hand, the pathological clones manage to take over the immune system by producing immuno-modulatory cytokines (IL-4, IL-10, and TGFβ) and growth factors (VEGF, TNFα, thrombopoietin, etc.) and modulating Treg cell activity. The pro/con Immune activity is certainly variable according to the stage of the disease. At the stage of clinical expression, MDS certainly is already winning against the immunosurveillance. Finally, the immune system itself can suffer from MDS progression due to central cytopenia, Treg accumulation, exhaustion of phagocytosis and cytotoxicity, repertoire skewing and possibly autoimmune disorders.

## Author Contributions

All authors listed have made substantial, direct, and intellectual contribution to the work and approved it for publication.

## Conflict of Interest Statement

The authors declare that the research was conducted in the absence of any commercial or financial relationships that could be construed as a potential conflict of interest.

## Abbreviations

AAM, alternatively activated macrophages; AML, acute myeloid leukemia; BM, bone marrow; CFU-GM, colony forming unit of granulocytes and monocytes; Cy, cyanine; Dim, diminished; EDTA, ethylene diamine tetra acetic acid, ESCCA, European Society for Clinical Cell Analysis; FCM, flow cytometry; FITC, fluorescein isothiocyanate; FSC, forward scatter; MdFI, median fluorescence intensity; MDS, myelodysplastic syndrome; MDSC, myeloid derived suppressive cells; MRD, minimal residual disease; PAMP, pathogen-associated molecular patterns; PB, peripheral blood; PE, phycoerythrin; ROS, reactive oxygen species; SSC, side scatter; TLR, toll like receptors; WHO, World Health Organization.
